# Naturally Occurring Allotropes of Carbon

**DOI:** 10.1021/acs.analchem.3c04662

**Published:** 2024-01-26

**Authors:** Zahra Farmani, Alessandro Vetere, Norbert Pfänder, Christian W. Lehmann, Wolfgang Schrader

**Affiliations:** † Max-Planck-Institut für Kohlenforschung, Kaiser-Wilhelm-Platz 1, 45470 Mülheim an der Ruhr, Germany; ‡ Max-Planck-Institut für Chemische Energiekonversion, Stiftstr. 34-36, 45470 Mülheim an der Ruhr, Germany

## Abstract

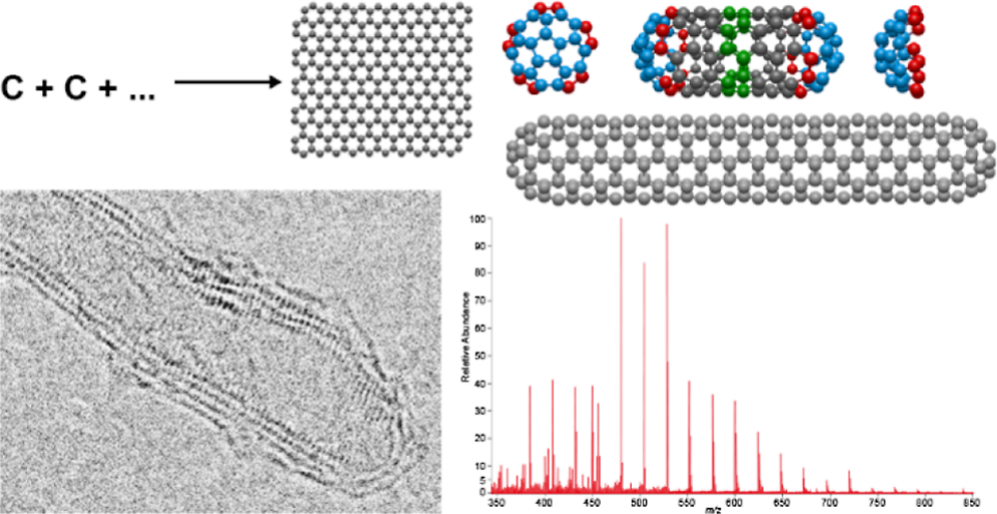

Carbon is one of the most important chemical elements, forming a wide range of important allotropes, ranging from diamond over graphite to nanostructural materials such as graphene, fullerenes, and carbon nanotubes (CNTs). Especially these nanomaterials play an important role in technology and are commonly formed in laborious synthetic processes that often are of high energy demand. Recently, fullerenes and their building blocks (buckybowls) have been found in natural fossil materials formed under geological conditions. The question arises of how diverse nature can be in forming different types of natural allotropes of carbon. This is investigated here, using modern analytical methods such as ultrahigh-resolution mass spectrometry and transmission electron microscopy, which facilitate a detailed understanding of the diversity of natural carbon allotropes. Large fullerenes, fullertubes, graphene sheets, and double- and multiwalled CNTs together with single-walled CNTs were detected in natural heavy fossil materials while theoretical calculations on the B3LYP/6-31G(d) level of theory using the ORCA software package support the findings.

## Introduction

Carbon is a fascinating element which can be referred to as the element of life in the periodic table.^1^ It is present in more than 95% of the known chemical compounds.^2^ Carbon-containing compounds play important roles in most of the natural processes in the universe; from the interstellar medium,^3^ unicellular organisms and the organic food chain to metabolic processes in the human body. Additionally, they act as the key components of many technological products in our modern life (e.g., synthetic polymers, pharmaceuticals, and energy-related materials). Such a great versatility of carbon is due to its unique ability to bind to almost all of the (electronegative and -positive) elements, with almost unlimited structural variety.^1^ Therefore, carbon-containing compounds possess a wide range of structural motifs and, consequently, physicochemical properties. Carbon allows a wide range of orbital hybridization (sp, sp^2^, and sp^3^) which enables covalent bonds to a number of different other elements but also to other carbon atoms.^4^ Hence, the allotropy of carbon is expected to encompass a wide range of different structures.^5^

In 1985, the discovery of a new carbon allotrope (C_60_ fullerene) initiated the advent of modern nanomaterials science.^6,7^ Since then, almost all nanomaterials are synthetic and are mostly carbon based. Shortly after, in 1991, with the discovery of another new allotrope of carbon (today known as nanotubes),^8^ carbon allotropy became an inseparable part of nanoscience. Until May 2017, at least 522 individual pure carbon structures (stable or metastable) were reported and yet more are expected to be discovered in the future.^9,10^ The most commonly known and well-studied natural allotropes of carbon are graphite and diamond. Additionally, naturally occurring fullerenes on earth were reported in several geological samples (fossil materials and bituminous rocks).^11,12^ To the best of our knowledge, there is no evident report about the natural occurrence of carbon nanotubes (CNTs), especially of single-walled CNTs (SWCNTs), up to now.^13^ Recently, CNTs (of unknown origin) were reported to have been found in sixth century B.C. potteries from India, which were referred to as “the oldest among the so far reported CNTs from ancient artifacts”.^14^

One of the oldest applied carbonaceous materials in the history of humans is natural asphalt (bitumen). There is evidence that Neanderthals, back in 40,000 B.C., were using bitumen as an adhesive material. Different archeological studies prove the use of bitumen by many ancient civilizations (e.g., Mesopotamians, Elam, and the Northwestern frontiers of the Indian subcontinent), which date back to 8900–10,000 B.C.^15^ In those eras, bitumen was used as mortar in construction building, for sealing and as a waterproofing agent, as well as an adhesive and also on domestic artifacts.^16,17^

Understanding such a complex and diverse system requires not only a single analytical method but also a finely tuned set of tools that allow a distinct understanding of the differences in structure. Most analytical methods, unfortunately, just give information about the bulk sample without details of the individual species.^18^ Here, in addition to ultrahigh resolution mass spectrometry (UHRMS), electron microscopic methods also allow a detailed understanding of the different carbon species and reveal new insights into naturally occurring carbon allotropes.

## Experimental Section

### Asphaltene Fraction Preparation

Two different crude oils, from different geographical origins, were used as samples. One sample was of American origin, while the other was a heavy crude oil whose origin is unknown to us. Six g of each sample were collected and their asphaltenes were precipitated using *n*-heptane (HPLC grade, Merck, Germany). A modified IP-143 method was used here.^19,20^ Briefly, 180 mL of *n*-heptane (30 mL/g of sample) was added and then mixed for 2 h to obtain a fully dispersed solution. The mixture was then cooled and stored in the dark overnight. The precipitates were filtered through filter paper (Whatman, type 589.3). The sample was rinsed with hot *n*-heptane and then the filtered sample together with the filter paper placed into a Soxhlet apparatus. To obtain a pure asphaltene fraction, the sample was then washed for 15 days using *n*-heptane solvent. Therefore, all possible traces of the maltene fraction should be fully removed from the asphaltene sample. Later the asphaltene fraction was extracted using toluene (HPLC grade, Merck, Germany). The collected fractions were evaporated to dryness and stored for further analysis.

### Electron Microscopy

To gain more details about possible nanocarbonaceous materials in the samples, they were analyzed using electron microscopy (EM). Scanning transmission electron microscopy (STEM) was performed using a Hitachi HD-2700, C_S_ corrected dedicated STEM, operated at 200 kV, Cold FEG, equipped with an energy dispersive X-ray spectrometer (EDAX Octane T Ultra W 200 mm^2^ SDD TEAM-Software).

### High-Resolution Mass Spectrometry

Mass spectra were recorded on a research-type Orbitrap Elite mass spectrometer (Thermo Scientific, Bremen, Germany) using electrospray (ESI(+)) ionization. The recorded mass range was *m*/*z* 200–1500, using spectral stitching (mass windows of 30 Da with 5 Da overlap), at a mass resolving power of *R* = 480,000 and 960,000 (fwhm at *m*/*z* 400).

The asphaltene sample was diluted in a toluene/methanol (1:1, v/v) mixture with a final concentration of 250 μg mL^–1^ and then analyzed with no further treatment by direct infusion with a flow rate of 5 μL min^–1^. The electrospray ionization was performed in positive mode at a spray voltage of 4.2 kV with a sheath gas flow of 7 arbitrary units. The flow rates of the auxiliary gas and sweep gas were set to 5 and 2 arbitrary units.

### MS Data Analysis

The acquired data were analyzed by Composer64 ver. 1.5.3 (Sierra Analytics, Inc., Modesto, CA, USA). For peak assignment, the following criteria were applied: H: 0–1000, C: 0–200, N: 0–3, O: 0–3, S: 0–3, DBE: 0–80 with a maximum mass error of 1 ppm.

The double bond equivalent (DBE) is used as an important parameter to compare the degree of aromaticity for different components. DBE refers to the number of ring closures and the number of double bonds within a molecule. This value can be calculated from the molecular formula (C_*c*_H_*h*_N_*n*_O_*o*_S_*s*_) of each individual assignment using the following equation: DBE = *c* – *h*/2 + *n*/2 + 1.

### Computational Details

All calculations were carried out using the ORCA 4.0 program package.^21,22^ All structures were optimized at the B3LYP/6-31G(d) level of theory.

## Results

### Fullerenes in Bitumen

Bitumen and other crude fossil fuel materials contain a high abundance of different carbonaceous compounds, often with a large number of other (hetero-) elements. For instance, conservative studies are estimating the presence of more than one million different chemical compounds, only in conventional crude oils.^23^ Bitumen is considered to be one of the heaviest crude oil materials and is expected to possess much higher chemical and structural complexity than conventional crudes. Due to such high complexity, less is known about its structural details or chemical constituents.^24^ This indicates that the real chemical understanding of such materials, which have been formed over millions of years under geological conditions, is still a big mystery. Despite the developments in analytical chemistry during the last decades, scientists succeeded in gaining only marginal knowledge about the structural entities or motifs in these samples.^25,26^ One of the major problems in analyzing these samples is that there is not one single analytical method available that can observe and analyze the whole mixture all at once. Part of the problem is the large variety of the different compounds ranging from highly volatile to nonvolatile, from nonpolar to polar, and from small single molecules up to solid mineral rock compounds. Additionally, it has been shown that a high sample complexity is sabotaging most of the analytical results due to suppression and discrimination effects.^27−31^ In this case, smaller fractions of a complex sample can reveal more molecular information than the whole mixture together. Thence, many bitumen and fossil crude material studies are performed on different fractions of these complex samples. Results of such analyses revealed the presence of many chemical compounds in different fractions of crude oils which resemble a “molecular form of a carbon allotrope”. Some examples are diamondoids resembling diamond,^32,33^ condensed polyaromatic hydrocarbons resembling graphene,^19,34^ and finally, in a recently published study,^32,35^ buckybowls resembling fullerenes. In the latter study, in addition to buckybowls, pristine fullerenes and quasi-fullerenes are also reported in the asphaltene fraction of a bitumen sample. This finding broke an almost century-old dogma that all aromatic moieties in crude oils are planar only.

**Figure 1:**
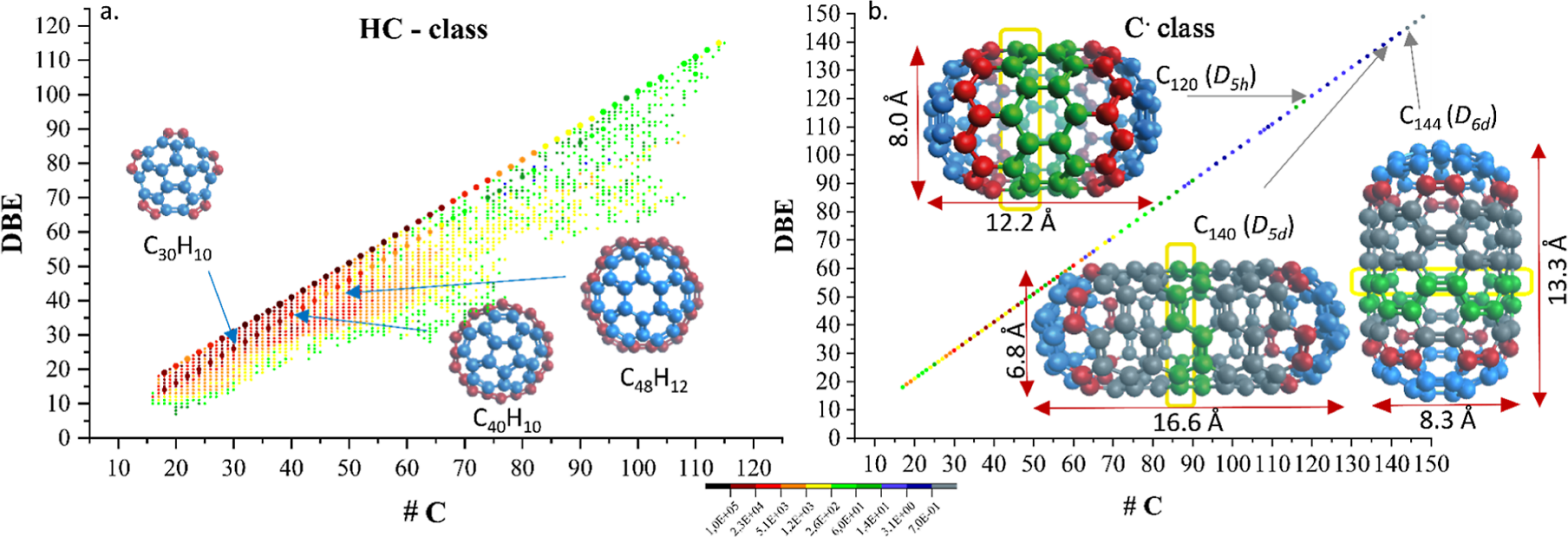
Mass spectrometric results from a bitumen sample. (a) Kendrick-type plot of the hydrocarbon class. Here, all signals are depicted that have been detected and assigned an elemental composition corresponding to an HC-class radical cation. Elemental compositions with C_30_H_10_ and C_40_H_10_ correlate to end-caps of fullertubes on a corannulene base while C_48_H_12_ correlates to an end-cap based on coronene. (b) Kendrick-type plot that shows the DBEs of the radical cation carbon class vs the number of carbon atoms. Each dot represents an assigned fullerene-related elemental composition, calculated from an accurate mass spectrometric signal. A wide range of fullerenes is observed in the asphaltene fraction of the natural asphalt sample (up to C_150_). The drastic decrease in the signal intensity of fullerenes larger than C_140_ indicates the limit of detection at this point as larger carbon compounds cannot be detected with the necessary sensitivity. Examples of higher fullerenes shown are related to their end-caps. Depending on the type of end-cap and symmetry, fullerenes of different lengths and diameters are formed.

UHRMS is often the method of choice for studying carbon-type materials^36^ and here data reveal a wide range of carbon-only type compounds which are naturally occurring fullerenes. These studies have been expanded here using UHRMS. In Figure 1, the data are summarized in Kendrick-type plots. While MS data reveal only the accurate elemental composition obtained from accurately measured signals in the MS spectrum, calculations on the DFT level of theory can reveal some insights into potential structures. Here, we used B3LYP/6-31G(d) level calculations with the Orca software package^21,22^ to better understand how those smaller fullerenes transform into longer fullertubes.^37−39^ The data in Figure 1a show the hydrocarbon-class compounds. Among a large number of hydrocarbons, some special compositions are in agreement with potential fullertube end-caps. Examples of structures for C_30_H_10_, C_40_H_10_, and C_48_H_12_ are shown. Those end-caps lead to different geometries of the fullertubes. While C_30_H_10_, which is based on a corannulene moiety, forms not only C_60_ it can also be the end-cap of fullertubes with *D*_5*d*_-symmetry. An example of a C_140_ compound is shown in Figure 1b. In addition to the end-caps, here a ring-type structure of ten alternating 6-membered rings (zigzag chain)^40^ forms the tube. The periodicity of the smallest segment in this symmetry is 10 carbon atoms (see the yellow box). Another type of end-cap is C_40_H_10_, which is also based on a corannulene moiety but leads to fullertubes with a *D*_5*h*_-symmetry with a C_120_ shown as an example in Figure 1b. This end-cap leads to fullertubes with a larger diameter (6.8 vs 8.0 Å) where ten six-membered rings are linearly condensed to form the ring. Here, the smallest periodic segment consists of 20 carbon atoms. The third symmetry shown here as an example is the *D*_6*d*_-symmetry with a C_144_ and a coronene as basic end-cap moiety (C_48_H_12_). This type of structure leads to different periodic segments. Although the ring structure is similar to the *D*_5*d*_-symmetry the ring contains 12 6-membered rings in a zigzag chain conformation, thus making it bigger (8.3 Å) with the smallest periodic element of 12 carbon atoms (see the yellow box in Figure 1b). Recent synthetic studies forming fullertubes lead to a number of different types of species.^41^ Still, the number of fullerenes detected here is higher, indicating that nature seems to be more diverse and lab synthesis may be more selective. While initial studies reported a range of fullerenes of up to C_110_ detected by mass spectrometry,^35^ this range has been expanded to masses of up to 1800 Da, corresponding to a C_150_ fullerene.

Mass spectrometric results revealed two things: First, a high number of fullertubes and fullerenes can be detected in fossil material, and second, mass spectrometry has a sensitivity limit at around C_150_ for those natural samples because no larger species could be detected. This can be the case because higher mass species cannot be measured by MS due to low volatility or the concentration of these compounds is just too low for detection. However, it can be envisioned, that, if such a high variety of carbon-only compounds can be detected by MS, many even larger allotropes of carbon exist, for whose detection a different analytical method is needed.

### STEM Studies of Natural Nanostructured Materials–Graphene Sheets

As shown in Figure 1, the sensitivity of mass spectrometry for larger carbon species is drastically decreased and reaches the limit of detection at around C_150_ on these natural materials. To better understand the structural identity of carbon species present in a bitumen, we have covered a wide range of analytical methods to gain any additional information, starting with infrared spectrometry, NMR, or different types of chromatography among others. The results were all together not reportable because the few carbon allotropes are competing during analysis with thousands of similar polyaromatic hydrocarbons, and here the complexity of the hydrocarbons won, limiting the range of analytical information that can be gained, while only bulk data are available.

**Figure 2:**
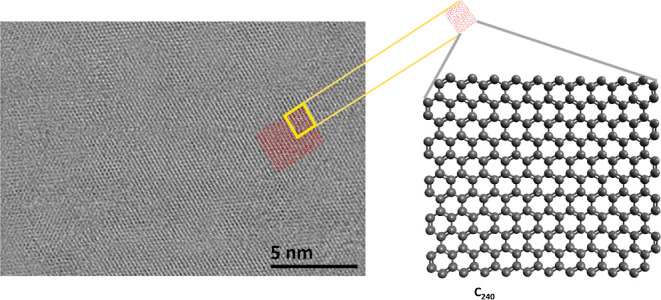
BF-STEM micrograph (left) revealing a graphene sheet obtained from the measurement of an asphaltene sample. The rectangular yellow box indicates a potential structural motif (in this case a 10 × 10 array) of 6-membered rings as shown on the right side, making it a C_240_ motif. The red area indicates a rectangle that combines 6 of those motifs, forming a structure that has a molecular mass above 17,000 Da (reconstructed image of BF-STEM micrograph obtained at 200 kV). For further details, see Experimental Section and Supporting Information. For a better understanding of the image, different model pictures were calculated and are shown in the Supporting Information.

On the other side, much of our knowledge about the structure of carbon allotropes and especially nanomaterials originates from studies using EM^42,43^ or X-ray diffraction (XRD).^44^ Most of the carbonaceous nanostructures (e.g., giant fullerenes or nanotubes) are often studied using SEM or TEM because with these methods it is possible—similar as in mass spectrometry—to focus on individual species.^42,43,45,46^ Therefore, in this study, the asphaltene fraction from a bitumen sample was analyzed, in addition to mass spectrometry, also using BF-STEM and SE-STEM. The main goal of these analyses was the detection of different naturally occurring CNTs or other allotropes of carbon (e.g., graphene) to better understand the complexity of carbon allotropes in natural mixtures. One significant example of extended structures, detected using EM, is depicted in Figure 2. This micrograph shows the defined and flat structure of a graphene sheet. For a better understanding of the approximate size, a model template array of 10 × 10 6-membered rings as shown on the right side in Figure 2 (a hypothetical molecule of C_240_) is overlaid onto the micrograph (see yellow box). Taking six units of this template makes the red rectangle, forming an area with a molecular mass above 17,000 Da. This indicates that the graphene flake in this micrograph has a molecular mass, which is exceeding 175,000 Da by far, explaining why mass spectrometry is no longer useful for these types of species.

### STEM Studies of Natural Nanostructured Materials—Folding Phenomena

**Figure 3:**
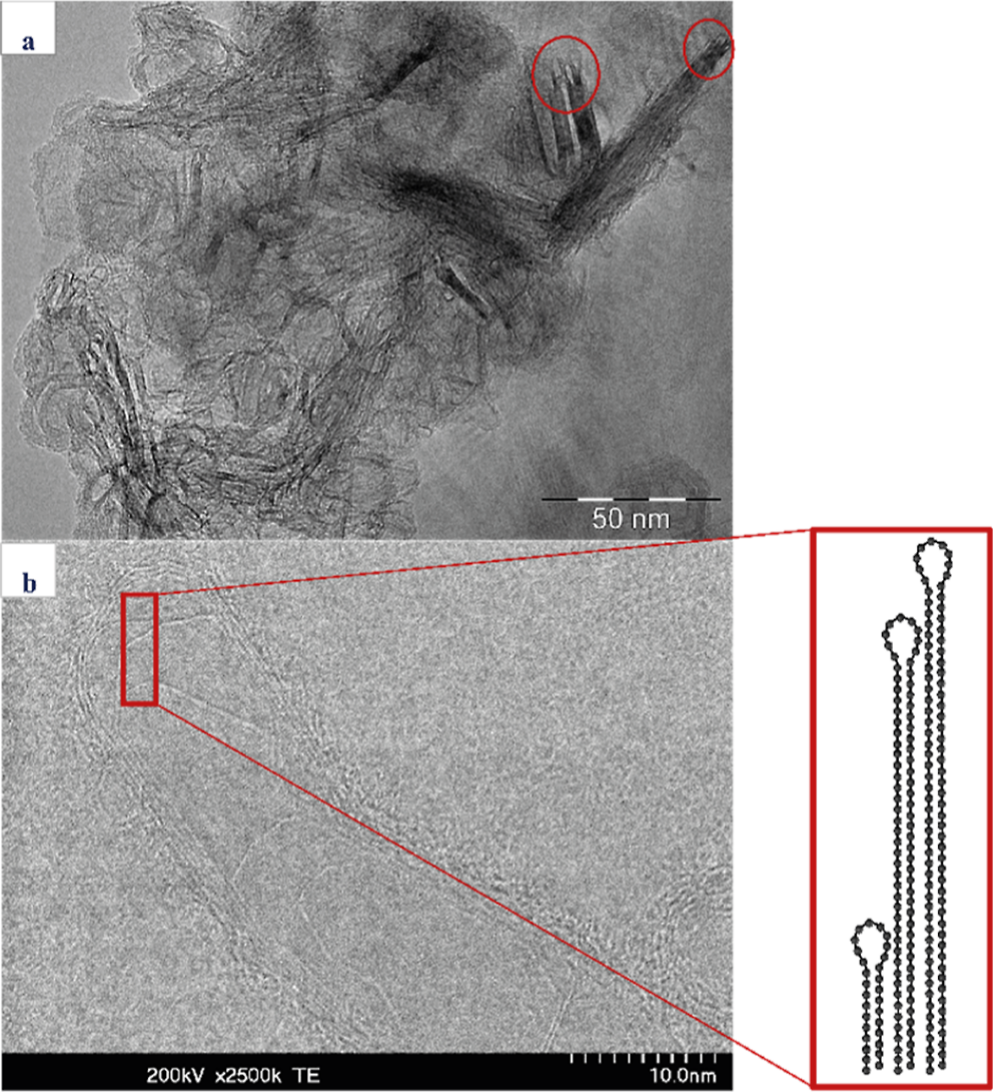
In graph a, the two red marked regions show the cross-sectional view of multiple bilayers of graphene sheets. Such bilayer images are reported for closed edges of graphene sheets.^1−3^ Graph (b) shows an overview of multiple bilayers of graphene sheets. The lighter color borders in this image indicate the voluminous regions known as the close edges of the graphene sheet. The cross-sectional view of the marked region is presented in a demonstrative model.

It has been reported that graphene sheets behave in a manner that they do not stay planar but tend to fold and buckle, which would lead to the formation of 3-dimensional folded structures.^47,48^Figure 3 shows a BF-STEM (bright-field-scanning transmission electron microscopy) micrograph of the analyzed sample. In this image, several parallel bilayers of graphene sheets are stacked on top of each other. Two marked regions in Figure 3a show a cross-sectional view of different bilayers of graphene sheets in the sample. According to the available literature, the voluminous area of the edges is the folding point of graphene sheets, which are known as “closed edges of graphene sheets”.^49−51^ In Figure 3b, the bilayer sheets are located perpendicular to the tilt axis of the camera. According to the aforementioned literature, the bright color edges are the closed edges of the graphene sheets. A model of the cross-sectional view of the selected region in Figure 3b is presented on the right side of the image. The closed edges of graphene sheets resemble the partial geometry of CNT structures.^49−51^

### STEM Studies of Natural Nanostructured Materials–Carbon Nano Tubes

**Figure 4:**
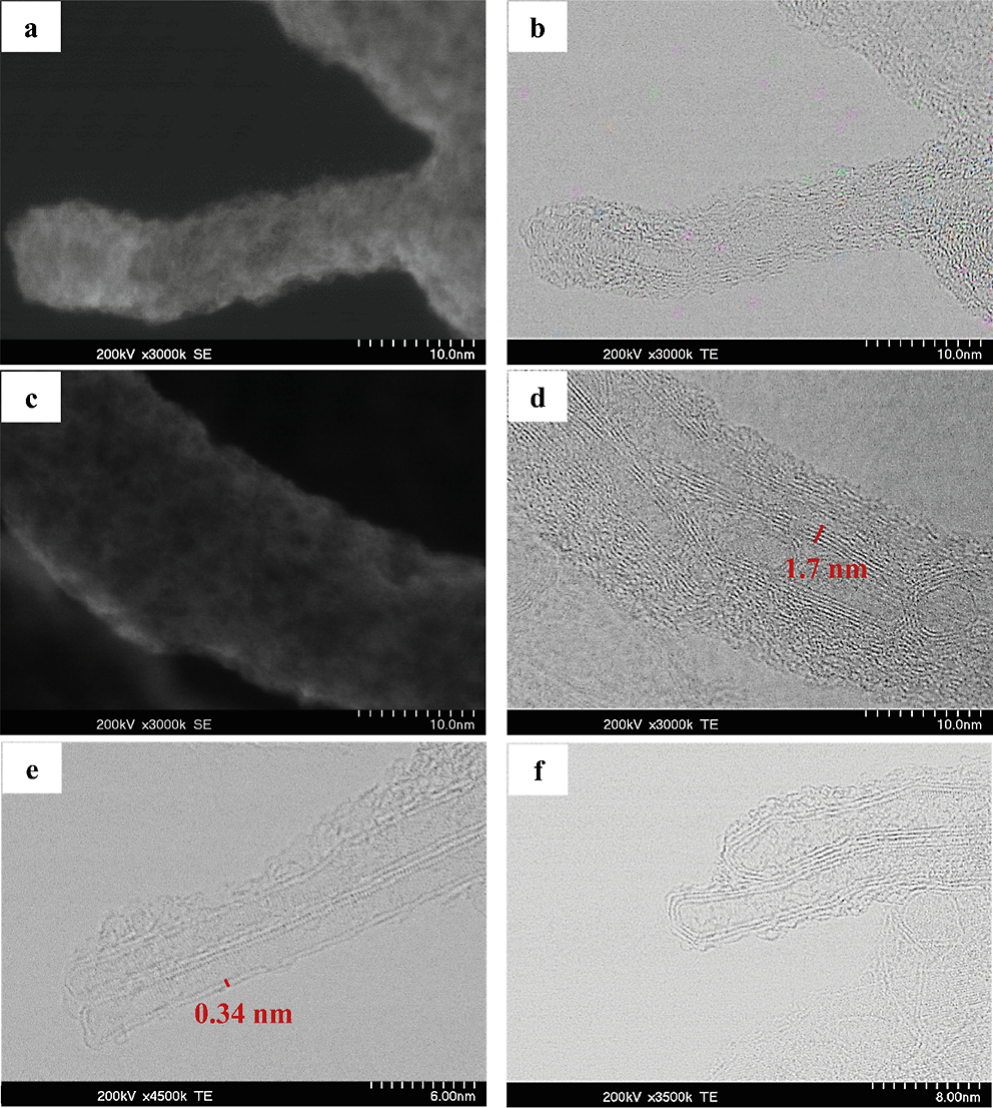
SE-STEM (a) and BF-STEM (b) micrographs of an MWCNT. The STEM image shows that the outer surface of the detected MWCNT is covered by amorphous carbon. The other set of SE_STEM (c) and BF-STEM (d) images show several MWCNTs forming a bundle, which is also covered with amorphous carbon on its outer area. The total distance between the inside and outside walls of an MWCNT in the image (d) is measured as 1.7 nm, which results from 0.34 nm spacing between 5 walls of the CNT. Images (e,f) show two DWCNTs and MWCNTs accordingly. The distance between two walls of DWCNT is measured as 0.34 nm.

Indeed, different EM analyses show the presence of other nanostructures, in addition to fullerenes and (folded) graphene sheets in the asphaltene sample. Some of these nanostructures include double-walled CNTs (DWCNTs) and multiwalled CNTs (MWCNTs) ranging from 3 up to 8 walls. A set of detected DWCNTs and MWCNTs is shown in Figure 4, where different tubes stick out of the carbonaceous material. In Figure 4a,c, the SEM images of CNTs are illustrated, while images (b) and (d) represent their corresponding TEM images. The SEM data show only the surface region of the tubes sticking out, while the TEM micrographs reveal the different multiwalled structures (DWCNTs and MWCNTs).

As shown in the STEM images, the outer surface areas of the depicted CNTs are covered with amorphous carbon. Figure 4e shows two DWCNTs that lay alongside each other. Here, the fine structure of the different walls allows us to measure the distance between two walls, which was determined to be 0.34 nm. The interwall distances of other MWCNTs were also measured and showed the same result. For instance, in Figure 4d, the distance between five walls of a MWCNT is measured to be 1.7 nm, which corresponds to the 0.34 nm spacing between four consecutive walls. The measured interwall distance in natural CNTs of this study (0.34 nm), agrees with the reported average value of interwall distances in CNT literature.^52,53^ These detected CNTs reveal new nanostructures in natural carbonaceous materials. As shown in the micrographs, CNTs are not uniform and form different varieties in terms of their morphology and association.

Some of the detected CNTs are rod shaped, while others are more curved and asymmetrical. Some are aligned together, whereas others are located angular to each other. Even the distribution of the CNTs throughout different regions of the sample is varying. Additionally, MWCNTs are observed to be diverse, in terms of their wall numbers (DWCNTs and MWCNTs).

### Details of Different Natural CNTs

DWCNTs are formed through the coaxial alignment of two SWCNTs. Consequently, they share many of the important characteristics of their single-walled counterparts (e.g., metallic and electrical properties), though due to the presence of a second wall, they possess additional advantages (e.g., mechanical and thermal robustness).^54,55^ They can be seen as the thinnest possible MWCNT. DWCNTs are less studied compared to the other CNTs. In recent years, more studies have focused on DWCNTs, due to their potential application for understanding interlayer interactions and electronic properties of CNTs.^56^ In the samples studied here, unlike the DWCNTs and MWCNTs, no SWCNTs were found to stick out from the bulk particle. This could be due to the fragility of single-walled structures toward physical and mechanical environmental factors, affecting a geological sample. Nevertheless, in these samples, several single-walled carbon nanostructures (such as giant fullerenes and SWCNTs) are observed to be either entrapped or embedded into larger structures (e.g., in between graphene sheets or inside of other CNTs).

**Figure 5:**
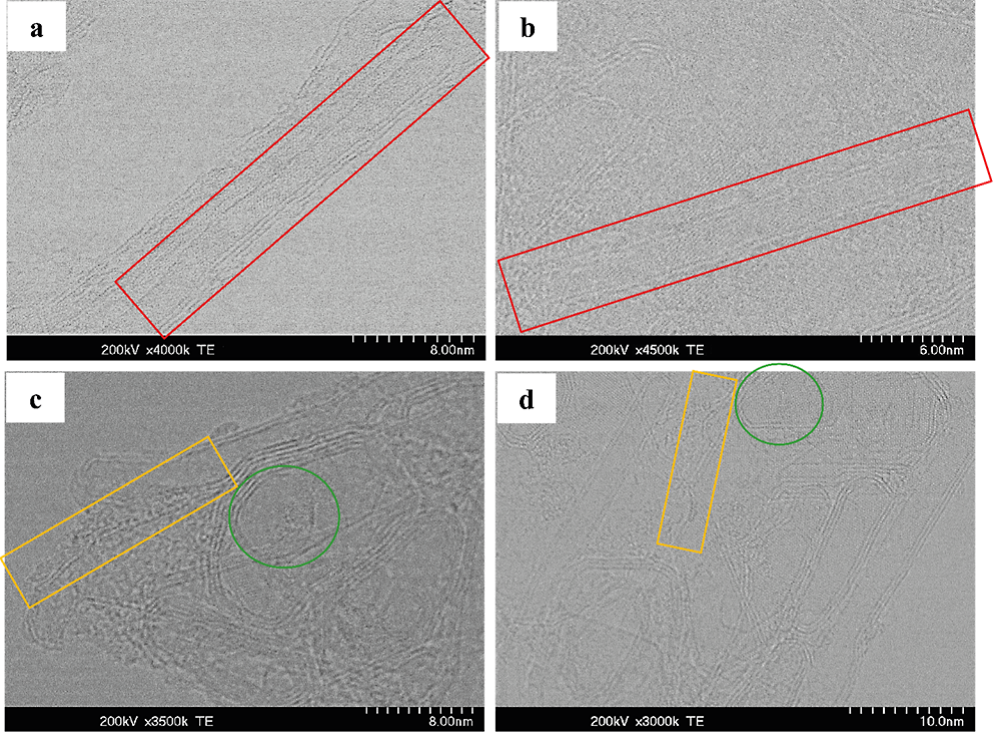
STEM micrographs of different asphaltene samples. Images (a,b) show the presence of SWCNTs with internal diameters of less than 2 nm (marked with red rectangles). These structures are always observed as either embedded inside other CNTs or embedded between other carbonaceous structures. Images (c,d) show the presence of another type of SWCNTs which according to the available literature are often observed in the form of bundles and have an internal diameter of 0.4 nm. Additionally, other single-walled structures similar to the one in the image (c) (marked with the green circle) are observed between other structures. These single-walled structures can potentially be giant fullerenes. Examples (a,b) are from an asphaltene obtained from a bitumen, while examples (c,d) are asphaltenes from a heavy crude oil.

Figure 5 depicts different varieties of single-walled nanostructures in different materials. Figure 5a,b shows the presence of SWCNTs encapsulated in other CNTs (marked in red) in a bitumen material. According to the literature, these entrapped SWCNTs can easily slide or rotate inside of MWCNTs, whereas they are well protected and preserved through the robustness and strong mechanical properties of those multiwalled structures.^57^

This explains how fragile single-walled nanostructures are preserved for thousands of years in bitumen (natural asphalts) as geological samples. Figure 5c,d shows an additional type of SWCNTs, ultrathin SWCNTs, embedded in between graphene sheets, raft-like graphite stripes, and MWCNTs, which have an internal diameter of 0.4 nm found in a heavy crude oil asphaltene. According to the literature, such SWCNT arrays are expected to be less stable and are only observed in embedded forms; in between graphite sheets, raft-like graphite stripes, and carbon onion-like structures.^58,59^ In general, CNTs come in three types of structures: zigzag, armchair, and chiral. These differ in their internal diameters.

The internal diameters of such ultrathin SWCNTs with zigzag, armchair, and chiral structures are reported to be 0.393, 0.407, and 0.414 nm.^58^ The end-caps of such ultrathin SWCNTs could be one-half of a C_20_ fullerene,^58,60−62^ which also was detected in these samples using mass spectrometry.^35^

These allotropes can be present in different crude oils ranging from medium to heavy to bitumen. The limit seems that the crude needs to have some amount of asphaltene content, which can already be found in medium crudes in some amounts.

## Conclusions

Among the different allotropes of carbon, to date, only fullerenes are reported to be naturally occurring in carbon-rich geological samples.^11,12,35^ MWCNTs are considered to have a high potential for natural occurrence as they are easily producible.^13^ But to date, their natural occurrence has been an open debate.

Bitumen—and especially its asphaltene fraction—is a carbon-rich and hydrogen-deficient natural material with a high presence in nature that contains a large number of different carbon-type compounds, which makes their structural understanding very difficult. Analytical methods that are not capable of selectively revealing molecular information on individual compounds in such a complex mixture as NMR,^18^ infrared spectroscopy, or others can only produce information on the bulk material. Here, only methods can help that allow the detection of individual compounds, such as high-resolution mass spectrometry and some selective types of EM.

In this study, several functional structures are detected in the asphaltene fraction of different crude oil samples that have not been reported before. It is worth noting that the asphaltene content plays an important role here. Naturally occurring CNTs of different types (MWCNTs, DWCNTs, SWCNTs, and ultrathin SWCNTs) are detected in these asphaltene samples using EM. Due to the extreme complexity of the samples, XRD analyses could not confirm these structures. Nevertheless, results of UHRMS detecting carbon-only structures bigger than C_100_ are the first indication for the presence of fullertubes, small CNT-like structures. Additionally, the results of STEM analyses show a wide range of different CNTs in these samples, which are comparable with some of the synthetic CNTs in the literature.

Laboratory synthesis of such nanostructures requires high amounts of energy (e.g., arc discharge, very high temperature), which does not exist in the natural geological environment of bitumen formation. Nonetheless, it is expected that nature possesses other driving factors for a different production route of these structures from that developed by scientists in laboratories. With reference to several studies, in the natural geological environment, parameters such as pressure, long reaction time, hydrothermal events,^63^ presence of clay minerals and metals (with catalytic activities)^64,65^ would be among the driving forces for the formation of such naturally occurring nanostructures. It has to be noted though that the amount of structured material cannot really be quantified due to missing methods for quantification. The data reveal that a very complex set of compounds is available, suggesting that the number of individual species is not very high.

With reference to the results of this study, the origin of the oldest reported CNTs in 600 B.C. potteries^14^ could now be explained by bitumen used as a sealing and waterproofing agent inside of those potteries. Here, our study can solve a mystery of the past.

## Supporting Information Available

Supporting material for STEM micrograph interpretation (PDF)
