# Supplementary material for: Naturally Occurring Allotropes of Carbon
Source: Anal Chem. 2024 Jan 26;96(7):2968–74. doi: 10.1021/acs.analchem.3c04662 (PMC10882575; doi:10.1021/acs.analchem.3c04662)

Supporting Information

Naturally occurring allotropes of carbon

Zahra Farmani,[1] Alessandro Vetere,[1] Norbert Pfänder,[1,2] Christian W. Lehmann,[1] Wolfgang Schrader[1]*

[1]Max-Planck-Institut für Kohlenforschung, Kaiser-Wilhelm-Platz 1, 45470 Mülheim an der Ruhr, Germany; email: wschrader@kofo.mpg.de
[2]Max-Planck-Institut für Chemische Energiekonversion, Stiftstr. 34-36, 45470 Mülheim an der Ruhr, Germany.

*Corresponding author. Email: wschrader@kofo.mpg.de

Content:

Micrograph Interpretation	S2
Figure S1	S3
Figure S2	S4
Figure S3	S5
Figure S4	S6
Figure S5	S7

Micrograph interpretation

The detection of graphene sheets embedded in the bitumen samples investigated by high-resolution, abberation corrected STEM is limited by the contrast transfer function (CTF) and the focussing plane selected during the scan for these non-planar areas of the sample.

Figure S1 shows an enlarged region containing an area of a hexagonally ordered structure. The inversion of contrast in this hexagonal pattern (bright dots and black dots) is clearly visible. The periodic spacing in the pattern amounts to 0.246 nm.

Figures S2 and S3 represent simulated hexagonal patterns with either grey or white circular objects spaced at 0.246 nm and the corresponding FFT diffractograms calculated using a Hanning Window type filter showing a diffraction spot distance of 4.71 nm^{-1}. In order to avoid streaks in the FFT image (due to sudden change of contrast at the edges of the 1024 x 1024 region of interest (ROI) shown in the left image) the image has rotated by few degrees to avoid lining up of the “holes” with the x-axis.

Figure S4 shows a model of a graphene sheet, with grey carbon atoms spaced at 1,42 Å from each other and a hexagonal unit cell with edges of 0.246 nm. The resulting FFT diffraction pattern shows the same spacing of reflections as observed in Figures S2b and S3b. In contrast a double layer of graphene (or a stack of graphene sheets as in graphite) is represented in Figure S5a. Again the in-plane distance between the carbon atoms is 1.42 Å, with every second atom sitting over the center of six-membered carbon ring. The corresponding FFT diffractogram for the resulting smaller unit cell, has a spacing of reflections of 8.18 nm^{-1}.

All model images have been generated using the crystal structure visualisation tool Diamond (Diamond Ver. 4.6.5, © 1997-2021, Crystal Impact GbR, Bonn, Germany). These images in TIFF-format have been imported into and FFT-patterns calculated by Digital Micrograph (Digital Micrograph Ver. 3.50.3584.0, © 1996-2021, Gatan Inc., Pleasanton, USA).

Fig. S1.

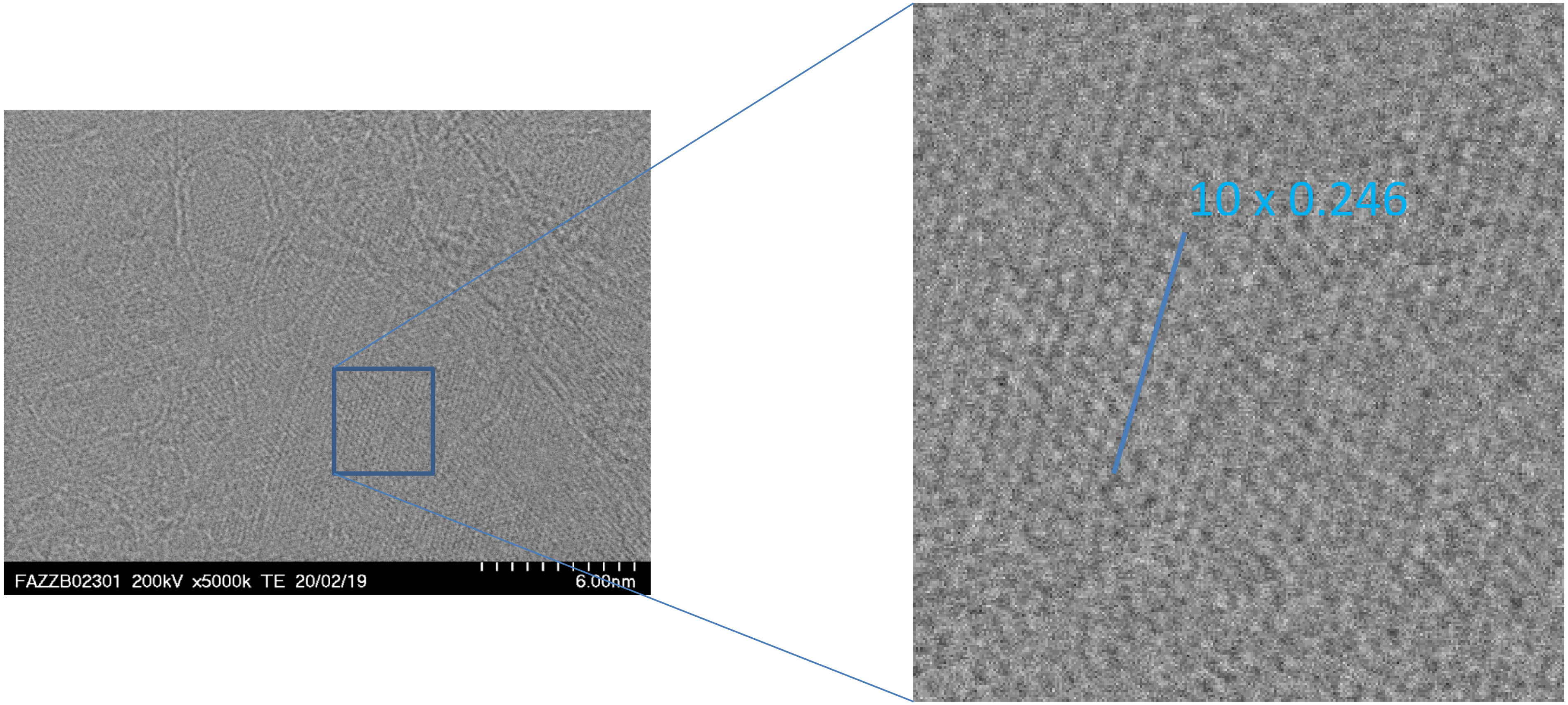

Fig. S2.

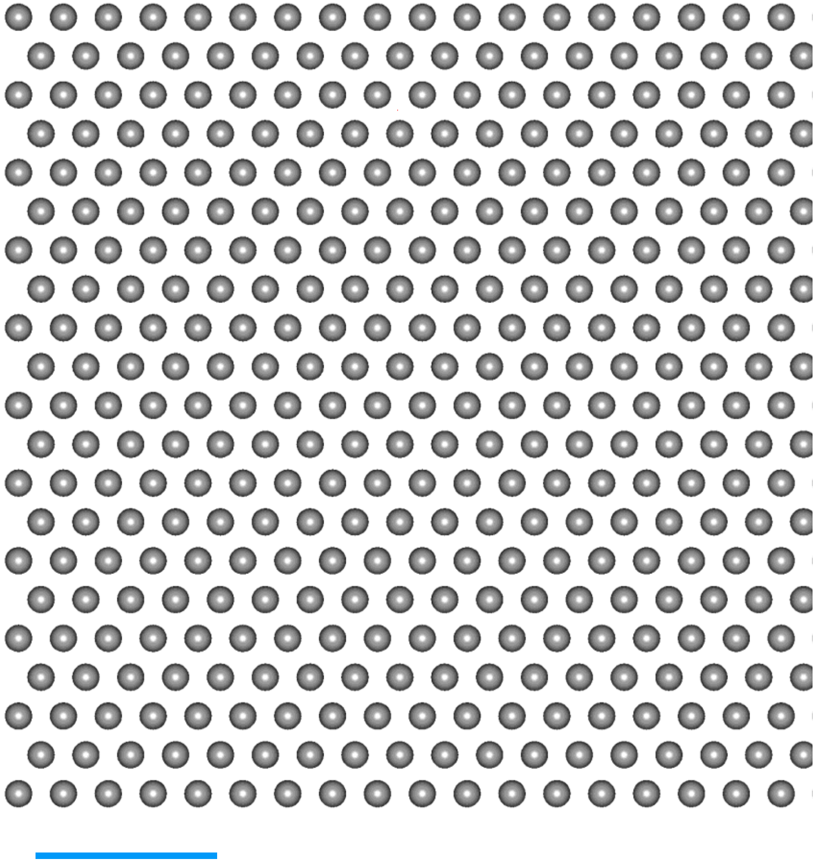

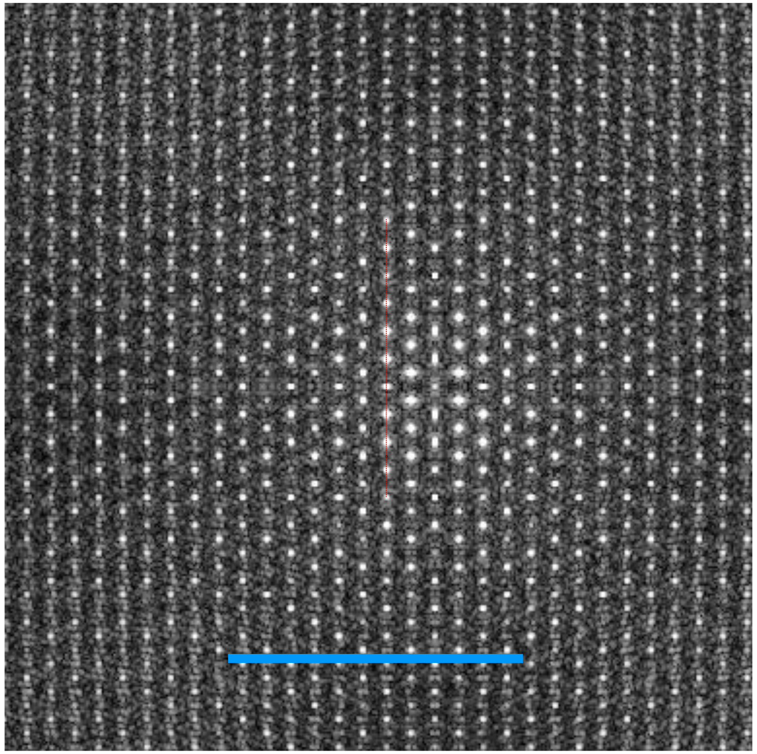

Fig. S3.

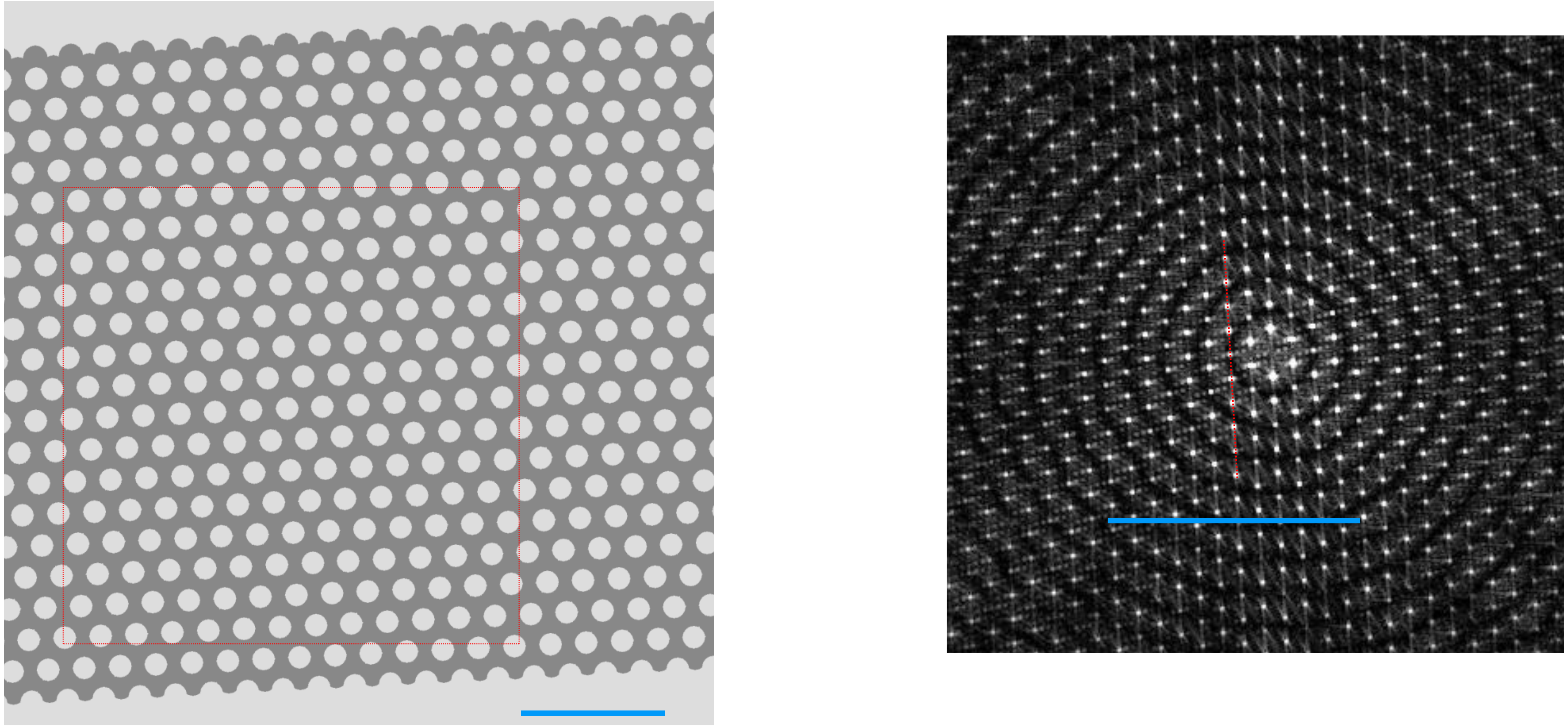

Fig. S4.

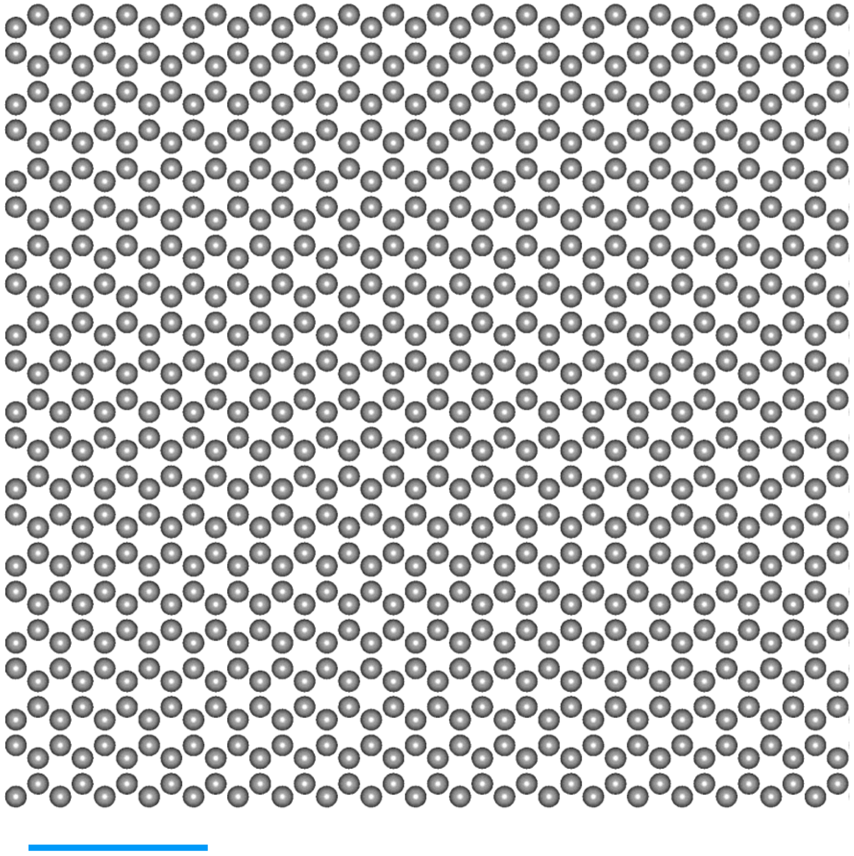

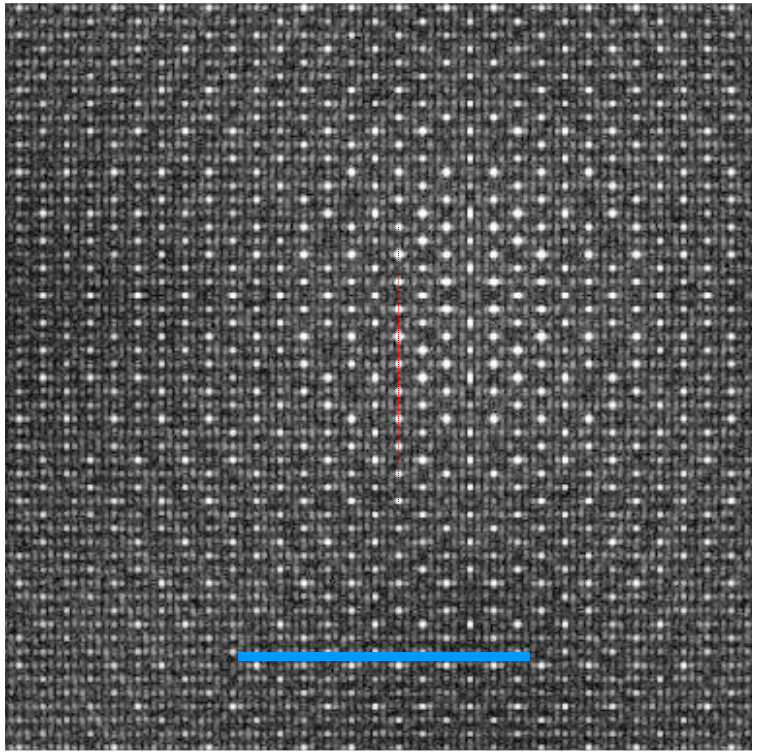

Fig. S5.

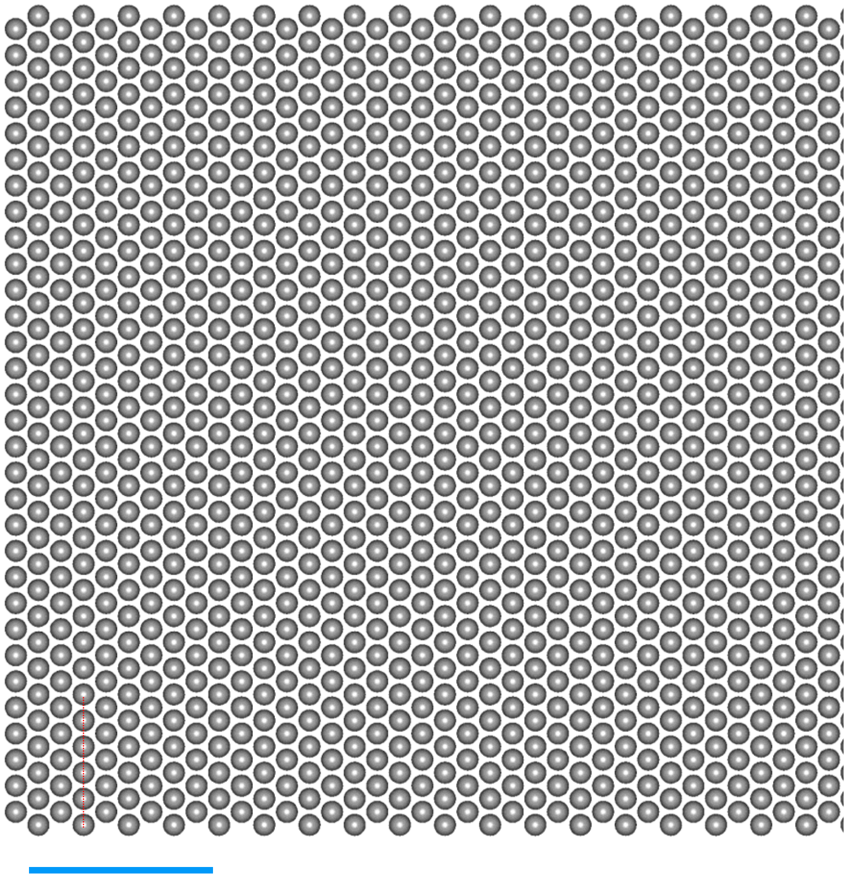

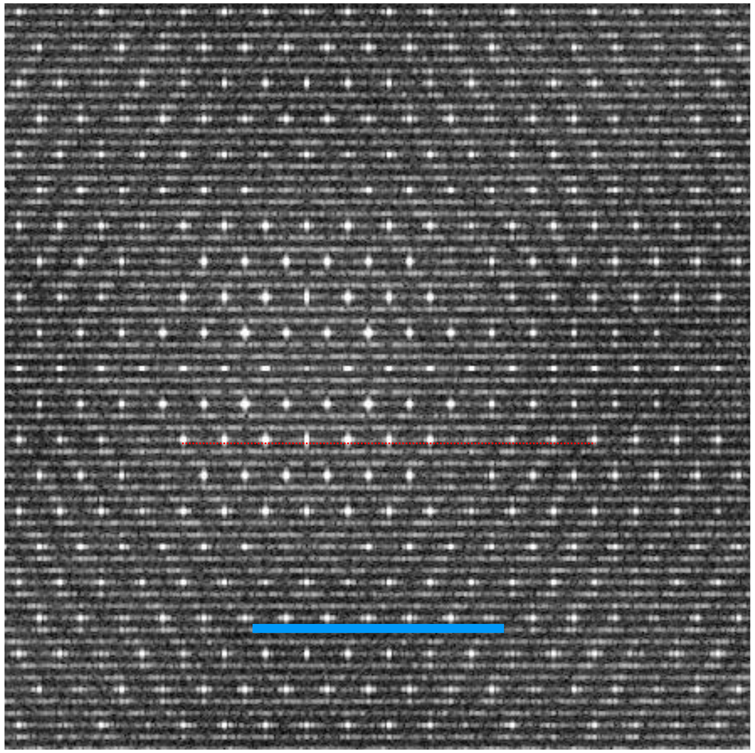

Supplement: Supplementary file 1 — ac3c04662_si_001.pdf [file ac3c04662_si_001.pdf]
